# SARS-CoV-2 Illumina GeNome Assembly Line (SIGNAL), a Snakemate workflow for rapid and bulk analysis of Illumina sequencing of SARS-CoV-2 genomes

**DOI:** 10.1093/nargab/lqae176

**Published:** 2024-12-18

**Authors:** Jalees A Nasir, Finlay Maguire, Kendrick M Smith, Emily M Panousis, Sheridan J C Baker, Patryk Aftanas, Amogelang R Raphenya, Brian P Alcock, Hassaan Maan, Natalie C Knox, Arinjay Banerjee, Karen Mossman, Bo Wang, Jared T Simpson, Robert A Kozak, Samira Mubareka, Andrew G McArthur

**Affiliations:** M.G. DeGroote Institute for Infectious Disease Research, McMaster University, 1280 Main Street West, Hamilton, Ontario, L8S 4K1, Canada; Department of Biochemistry and Biomedical Sciences, McMaster University, 1280 Main Street West, Hamilton, Ontario, L8S 4K1, Canada; Faculty of Computer Science, Dalhousie University, 6050 University Avenue, Halifax, Nova Scotia, B3H 1W5, Canada; Institute for Comparative Genomics, Dalhousie University, 5850 College Street, Halifax, Nova Scotia, B3H 4R2, Canada; Department of Community Health & Epidemiology, Dalhousie University, 5790 University Avenue, Halifax, Nova Scotia, B3H 1V7, Canada; Perimeter Institute for Theoretical Physics, 31 Caroline Street North, Waterloo, Ontario, N2L 2Y5, Canada; M.G. DeGroote Institute for Infectious Disease Research, McMaster University, 1280 Main Street West, Hamilton, Ontario, L8S 4K1, Canada; Department of Biochemistry and Biomedical Sciences, McMaster University, 1280 Main Street West, Hamilton, Ontario, L8S 4K1, Canada; M.G. DeGroote Institute for Infectious Disease Research, McMaster University, 1280 Main Street West, Hamilton, Ontario, L8S 4K1, Canada; Department of Biochemistry and Biomedical Sciences, McMaster University, 1280 Main Street West, Hamilton, Ontario, L8S 4K1, Canada; Division of Microbiology, Department of Laboratory Medicine and Molecular Diagnostics, Sunnybrook Health Sciences Centre, 2075 Bayview Avenue, Toronto, Ontario, M4N 3M5, Canada; M.G. DeGroote Institute for Infectious Disease Research, McMaster University, 1280 Main Street West, Hamilton, Ontario, L8S 4K1, Canada; Department of Biochemistry and Biomedical Sciences, McMaster University, 1280 Main Street West, Hamilton, Ontario, L8S 4K1, Canada; M.G. DeGroote Institute for Infectious Disease Research, McMaster University, 1280 Main Street West, Hamilton, Ontario, L8S 4K1, Canada; Department of Biochemistry and Biomedical Sciences, McMaster University, 1280 Main Street West, Hamilton, Ontario, L8S 4K1, Canada; Peter Munk Cardiac Centre, University Health Network, 585 University Avenue, Toronto, Ontario, M5G 2N2, Canada; National Microbiology Laboratory, Public Health Agency of Canada, 1015 Arlington Street, Winnipeg, Manitoba, R3G 1G1, Canada; Department of Medical Microbiology and Infectious Diseases, University of Manitoba, 745 Bannatyne Avenue, Winnipeg, Manitoba, R3E 0J9, Canada; M.G. DeGroote Institute for Infectious Disease Research, McMaster University, 1280 Main Street West, Hamilton, Ontario, L8S 4K1, Canada; Department of Veterinary Microbiology, Vaccine and Infectious Disease Organization, University of Saskatchewan, 120 Veterinary Road, Saskatoon, Saskatchewan, S7N 5E3, Canada; M.G. DeGroote Institute for Infectious Disease Research, McMaster University, 1280 Main Street West, Hamilton, Ontario, L8S 4K1, Canada; Department of Medicine, McMaster University, 1280 Main Street West, Hamilton, Ontario, L8S 4K1, Canada; McMaster Immunology Research Centre, McMaster University, 1280 Main Street West, Hamilton, Ontario, L8S 4K1, Canada; Peter Munk Cardiac Centre, University Health Network, 585 University Avenue, Toronto, Ontario, M5G 2N2, Canada; Department of Medical Biophysics, University of Toronto, 101 College Street, Toronto, Ontario, M5G 1L7, Canada; Vector Institute for Artificial Intelligence, 661 University Avenue, Toronto, Ontario, M5G 1M1, Canada; Ontario Institute for Cancer Research, 661 University Avenue, Toronto, Ontario, M5G 0A3, Canada; Department of Computer Science, University of Toronto, 40 St George Street, Toronto, Ontario, M5S 2E4, Canada; Department of Molecular Genetics, University of Toronto, 1 King's College Circle, Toronto, Ontario, M5S 1A8, Canada; Division of Microbiology, Department of Laboratory Medicine and Molecular Diagnostics, Sunnybrook Health Sciences Centre, 2075 Bayview Avenue, Toronto, Ontario, M4N 3M5, Canada; Department of Laboratory Medicine and Pathobiology, University of Toronto, 1 King's College Circle, Toronto, Ontario, M5S 1A8, Canada; Division of Microbiology, Department of Laboratory Medicine and Molecular Diagnostics, Sunnybrook Health Sciences Centre, 2075 Bayview Avenue, Toronto, Ontario, M4N 3M5, Canada; Department of Laboratory Medicine and Pathobiology, University of Toronto, 1 King's College Circle, Toronto, Ontario, M5S 1A8, Canada; M.G. DeGroote Institute for Infectious Disease Research, McMaster University, 1280 Main Street West, Hamilton, Ontario, L8S 4K1, Canada; Department of Biochemistry and Biomedical Sciences, McMaster University, 1280 Main Street West, Hamilton, Ontario, L8S 4K1, Canada

## Abstract

The incorporation of sequencing technologies in frontline and public health healthcare settings was vital in developing virus surveillance programs during the Coronavirus Disease 2019 (COVID-19) pandemic caused by transmission of the severe acute respiratory syndrome coronavirus 2 (SARS-CoV-2). However, increased data acquisition poses challenges for both rapid and accurate analyses. To overcome these hurdles, we developed the SARS-CoV-2 Illumina GeNome Assembly Line (SIGNAL) for quick bulk analyses of Illumina short-read sequencing data. SIGNAL is a Snakemake workflow that seamlessly manages parallel tasks to process large volumes of sequencing data. A series of outputs are generated, including consensus genomes, variant calls, lineage assessments and identified variants of concern (VOCs). Compared to other existing SARS-CoV-2 sequencing workflows, SIGNAL is one of the fastest-performing analysis tools while maintaining high accuracy. The source code is publicly available (github.com/jaleezyy/covid-19-signal) and is optimized to run on various systems, with software compatibility and resource management all handled within the workflow. Overall, SIGNAL illustrated its capacity for high-volume analyses through several contributions to publicly funded government public health surveillance programs and can be a valuable tool for continuing SARS-CoV-2 Illumina sequencing efforts and will inform the development of similar strategies for rapid viral sequence assessment.

## Introduction

The emergence of the novel betacoronavirus severe acute respiratory syndrome coronavirus 2 (SARS-CoV-2) (1) triggered a pressing need for genomic surveillance. The ability to detect pathogens, track transmission and scan for genomic mutations driving changes in function or transmissibility allowed for appropriate public health responses and aided development of therapeutics and vaccines. Comprehensive testing and surveillance programs generated many large volumes of samples from which the viral genomes could be sequenced to track genomic variants circulating in the community. However, this came with the challenges of ever-increasing data volume and demand for rapid turn-around times. Second-generation short-read NGS instruments like Illumina and third-generation long-read sequencing instruments like Nanopore allow for 100s of samples to be sequenced in a single run, opening the possibly for real-time genomic surveillance (2). Overall, outside of regional or national reference centers, DNA sequencers are uncommon within clinical microbiology laboratories due to additional technological, computational and educational requirements that add costs to already limited budgets (3). Technological requirements include sequencing instruments and reagents, alongside capacity to store and process sequencing data. Computational requirements include maintenance of technological infrastructure such as high-performance computing, data management and ever-growing storage costs. Finally, educational requirements include training personnel to operate sequencing equipment, advanced software to process, interpret and report output from the sequencers.

With sequencing technologies becoming more accessible and added investment to healthcare infrastructure due to the Coronavirus Disease 2019 (COVID-19) pandemic, both short-read and long-read technologies are being integrated into healthcare system whole genome sequencing programs. Yet, in the event of a large-scale outbreak cases rise exponentially, so quicker analyses allow for a more rapid and effective response. Here we present the SARS-CoV-2 Illumina GeNome Assembly Line (SIGNAL), a comprehensive workflow built to support regional and national SARS-CoV-2 Illumina-based sequencing efforts throughout Canada and beyond during the COVID-19 pandemic. Using Snakemake (a workflow manager extension of the Python language to provide seamless support for analysis of large numbers of samples on high-performance computers) and Conda (a package manager and environment management system) (4), a number of bioinformatic steps are run in parallel to provide a SARS-CoV-2 consensus genome sequences using reference-based assembly, identification of complex variants, sensitively remove patient genomic data and report the SARS-CoV-2 lineage for a given sample while flagging potential variants of concern (VOCs). A recent assessment (5) of SARS-CoV-2 assembly workflows found SIGNAL to have a 10-fold faster performance than the next fastest workflow, with negligible differences in consensus sequence prediction accuracy compared to other reference-guided workflows. This observed performance is reflective of SIGNAL’s initial aim to support large scale sequencing of SARS-CoV-2 samples during the peak of COVID-19 waves. In fact, during the first wave of SARS-CoV-2 infections in Canada, SIGNAL was responsible for ∼50% of the Province of Ontario’s and ∼20% of Canada’s genome sequences shared internationally on GISAID and to date SIGNAL has been used as the standard Illumina workflow at the Public Health Agency of Canada (PHAC). Here we describe the workflow, its component parts, its assumptions and its output.

## Materials and methods

### Installation and dependencies

#### Installation and configuration

Installation of SIGNAL requires Python 3 or later, including Conda (or Mamba) to obtain Snakemake (4) and Python Data Analysis Library (pandas) packages (6), after which a central executable script, *signalexe.py*, becomes usable to access all functions of SIGNAL (Supplementary Figure S1). As SIGNAL is a Snakemake workflow, a configuration file is required to provide necessary parameters, including paths to the necessary reference files and databases. For each run, the configuration file, *config.yaml* and file *sample_table.csv*, which lists the sample identifiers (generated using the filename) and file paths to paired R1 and R2 FASTQ files, must also be provided. These configuration and sample files can be created by hand from examples available in the SIGNAL GitHub repo or by use of the *signalexe.py –config_only* command.

#### Reference sequence download and databases

The required dependency reference files and databases can be obtained through a single-run flag, *signalexe.py –dependencies*. A National Center for Biotechnology Information (NCBI) accession identifier is required to download the necessary GenBank (.gbk), General Feature Format version 3 (.gff3) and FASTA files from the NCBI Nucleotide databases. By default, the first SARS-CoV-2 genome sequence from Wuhan, China (MN908947.3) is used as the reference genome sequence. The GRCh38 human genome reference is also downloaded from the NCBI genomes database. For Kraken2 (7), SIGNAL downloads the premade viral database containing up to date virus sequences through developer provided links (https://benlangmead.github.io/aws-indexes/k2), instead of the standard database generation step, to maintain quick performance.

#### Per-rule Conda environments

Each step within the SIGNAL workflow is defined as a Snakemake rule. Each rule sets both the software commands and required parameters to process input and intermediate data. Certain software versions are incompatible with one another, leading to errors in processing. To address these potential errors, each rule activates its own software environment when the rule is invoked. The separation of controlled software environments ensures that minimal software incompatibilities exist, and specific versions of software are used for reproducible analyses. Each environment can be created in advance using the *signalexe.py install* command. The specific versions of software used by each step in SIGNAL are outlined on SIGNAL’s GitHub page.

### Executing SIGNAL

#### General execution of signalexe.Py

For data analysis, SIGNAL expects one or a combination of three possible flags: ‘all,’ ‘postprocess’ or ‘ncov_tools.’ The command *signalexe.py all* is the main function of SIGNAL, performing all assembly and variant calling functions for all samples described in the sample configuration file *sample_table.csv*. The command *signalexe.py postprocess* provides per-sample and cumulative summaries of *signalexe.py all* results, while the command *signalexe.py ncov_tools* performs an independent analysis of data, consensus quality, and contamination using a third-party quality control package, *ncov-tools* (https://github.com/jts/ncov-tools). Upon execution of *signalexe.py*, Snakemake manages individual Conda dependencies for each rule in the workflow, installing and updating as needed should the per-rule Conda environments not be previously generated via *signalexe.py install*. Each sample is thus analyzed in parallel, with controlled Conda environments activated to allow execution of each step as required by the individual rule. As such, SIGNAL allows for maximum utilization of high-performance computing for a fast turn-around time.

### SIGNAL *all*

#### Raw input handling

The initial steps of *signalexe.py all* organize input files. Using the provided *config.yaml* file, SIGNAL locates the corresponding *sample_table.csv* file to determine the location of input FASTQ sequencing files and their sample identifier. Given the generally large file size of raw FASTQ sequencing files, SIGNAL symbolically links paired FASTQs within the SIGNAL results directory. This allows easy access to raw data for processing without compromising storage space. In some instances, multiples of paired FASTQs are generated during sequencing or resequencing for individual samples. These replicates will share the same sample identifier upon generation of *sample_table.csv*. To handle these replicate FASTQ pairs, SIGNAL concatenates and sorts the individual R1 and R2 reads with matching sample identifiers and known individual file paths to merge multiples into a single pair of FASTQ reads. All paired FASTQs, including subsequent trimmed pairs, are processed through FASTQC (http://www.bioinformatics.babraham.ac.uk/projects/fastqc) for quality assessment of the sequencing reads, with PDF and HTML output viewable and processable, respectively.

#### Removal of human reads

Even with amplicon- or bait-enrichment, sequencing clinical samples results in human, bacterial, and non-SARS-CoV-2-virus nucleotide content being sequenced alongside the target SARS-CoV-2. Not only do these anomalies in raw data increase the amount of background noise, but they also introduce patient privacy and health information considerations (impacting open data sharing). Prior to any analytical work for SARS-CoV-2, SIGNAL includes a step to remove human host derived DNA sequences. Reads are competitively mapped to a composite index generated from the human genome (GRCh38) and the reference SARS-CoV-2 genome (MN908947.3) using the Burrows-Wheeler Aligner's maximal exact matches algorithm (BWA-MEM) (8). This is then filtered to generate a set of de-hosted paired FASTQ only containing reads that either do not map to the human genome or map more strongly to the SARS-CoV-2 reference genome than the human genome. This enables open sharing of the most complete possible ‘raw’ sequencing data (vital for reproducibility) without infringing on individual human privacy.

#### Adapter trimming

During the library preparation step, amplification of viral nucleic acid is performed using SARS-CoV-2 specific polymerase chain reaction (PCR) primers and sequencing adapters, for example, the ARTIC4 primer set (https://github.com/artic-network/primer-schemes). This amplification step ensures that most of the material processed through a sequencer is virus material. However, the adapter sequences themselves are artificial sequences that add additional noise and reduce the overall accuracy of later assembly steps. For the standard Illumina adapters, we implement trim_galore (https://github.com/FelixKrueger/TrimGalore) as the first trimming step using user-defined minimum quality and read length thresholds to remove low-quality reads. A custom filter built in Python is applied afterwards to remove specific sequences corresponding to additional residual adapters not removed through trim_galore. The trimmed reads are mapped back to the reference SARS-CoV-2 genome and the iVar (9) trim function is used with a list of primer regions to remove additional artificial sequences. The final output of this step is a set of paired FASTQ files containing reads without sequencing primers and adapters.

#### Reference-based assembly and variant calling using iVar and Freebayes

SIGNAL uses two algorithms for variant calling and consensus generation for sequenced samples: iVar (9) and FreeBayes (10). From processed reads, a consensus FASTA sequence is generated by these tools based on read coverage and consensus at each position. The length of the final consensus sequence is equal to that of the reference genome, with unknown positions due to insufficient evidence labeled with ‘N’ characters. However, one of the critical junctions within SIGNAL is the ability to identify specific mutations that differ from the reference genome sequence (MN908947.3). Initial iterations of SIGNAL used the iVar variants function to perform variant calling, which identifies single-nucleotide variants (SNVs) using an alignment-based approach where read alignment against the reference sequence is performed using BWA-MEM and the resulting alignment file is processed through samtools (mpileup) (11) prior to iVar variant calling. Mutational differences across nucleotide positions are listed as variants in a tab-delimited output file. However, an uncommon but recurring issue with alignment-based variant calling through iVar was observed (https://github.com/andersen-lab/ivar/issues/85), whereby reads surrounding called indels were not realigned to account for the indel, which skewed coverage in the surrounding region and resulted in spurious variant calls in these regions. To remedy this, we incorporated FreeBayes (10), a haplotype-based variant caller with indel realignment that can better resolve these small variations, to run alongside the existing iVar variant caller. Users preferring to use iVar exclusively for SARS-CoV-2 consensus genome sequence generation can use the *–remove-freebayes* flag to exclude running of FreeBayes by SIGNAL. This selection has the benefit of faster performance, but with the reduced accuracy outlined above which may impact downstream lineage assignment. By default, both iVar and FreeBayes are run, and consensus sequences are compared to one another on a per-nucleotide basis.

#### Breseq and minor variants

The examination of individual mutations relative to the reference genome sequence (MN908947.3) is one of the most critical steps in the analysis of SARS-CoV-2. These specific combinations of mutations allow identification of the specific variant for a given sample and place it within the evolutionary context of other known sequences. Minor variants are mutations that make up a smaller proportion of sequencing reads within a sample, which may reflect a mixed population of virus within a sample or possible contamination. The reporting of minor variants is infrequent due to their lower prevalence in sequencing data and is often overlooked as we examine more prevalent combinations of mutations, referred to as major variants. These major variants are often the only ones reported across shared consensus genome sequences and thus, potentially relevant variants of interest (VOIs) may be unnoticed. Although iVar and FreeBayes variant calling outputs contain information about many of these minor variants, by default they will not call very low frequency variants and interpretation of these outputs can be technically challenging. Therefore, SIGNAL can also optionally use BreSeq (12) to sensitively identify low-frequency variants and generate an accessible human-readable report of all detected major and minor variants for each position of the SARS-CoV-2 genome. User-defined thresholds or default parameters *(–polymorphism-minimum-variant-coverage-each-strand 2 –polymorphism-frequency-cutoff 0.05)*aid in the reporting of minor variants, allowing users to differentiate findings from sequencing error or noise. This is a time-consuming step relative to the rest of the overall SIGNAL workflow so is only optionally included using the *–add-breseq* flag.

#### Coverage and other statistics

Depth of coverage for each nucleotide position is calculated using BEDTools (*genomecov -d*) (12) using only the sequencing reads included in consensus generation. The number of aligned reads is counted per nucleotide position and plotted using matplotlib (13). Kraken2 (7) is used for taxonomic identification, calculating the overall percentage of SARS-CoV-2 specific sequencing reads within the sample and allowing users to troubleshoot protocols should non-SARS-CoV-2 virus reads remain. The standard output for Kraken2 is provided as a text file detailing the entire taxonomic breakdown of all reads, SARS-CoV-2 or otherwise. QUAST (14) is also used for quality control assessment of the assembled consensus sequence.

#### PANGOLIN and NextClade lineage assignments

Beginning January 2021, the World Health Organization (WHO) began categorizing the presence of specific mutation combinations that corresponded to the potential or demonstratable increase in transmissibility and/or virulence. These labels included VOIs or VOCs. Prior to this, in the growing effort to track the evolution of SARS-CoV-2, the Phylogenetic Assignment of Named Global Outbreak Lineages (PANGOLIN) (15) framework was developed with distinct lineage names assigned to finely-grained epidemiologically relevant phylogenetic clusters (e.g. a specific outbreak or introduction) to aid with regional/national outbreak investigations and surveillance. As evidence grew that lineage B.1.1.7 involved (16–18) increased transmissibility across multiple populations, the WHO created its own labels for clearer communication about variants of major public health importance using the Greek alphabet. Consequently, the VOC with PANGO lineage B.1.1.7 was given the Alpha variant label by the WHO. The NextClade platform (19) was additionally developed to track the evolution of SARS-CoV-2 at a broader scale using NextStrain’s nomenclature system and to provide interactive phylogenetic analyses. As such, SIGNAL uses both PANGOLIN and NextClade to predict lineage/clade assignments describing the specific set of mutations/phylogenetic position of a given sample (i.e. PANGO lineage) alongside the sample’s phylogenetic placement relative to all other submitted sequences (i.e. NextClade assignment). Upon execution, SIGNAL automatically updates to the latest PANGOLIN and NextClade versions, along with required dependencies and databases, but users can optionally select specific versions of both software and dependencies for reproducibility (or when facing issues with external internet access, e.g., on compute nodes in high-performance computing facilities). The output from both tools is summarized in a tab-delimited file where each sample has its PANGO lineage, NextClade assignment, and associated QC results (with separate files for iVar and FreeBayes consensus sequences and variant calling information).

### SIGNAL postprocess

#### Summarizing across samples

Upon completion of *signalexe.py all*, a separate post-process step can be executed via *signalexe.py postprocess* to summarize the key findings from a given SIGNAL run, both on a per-sample basis and as overall summation. This summation step is separated from the *signalexe.py all* workflow due to file handling errors that can arise should any sample fail any steps (placeholder values are used in place of absent output). All post-processing steps dynamically update reports based on whether output exists for optional steps of the SIGNAL workflow, e.g. if FreeBayes is skipped, then only iVar consensus sequence and variant calling statistics are reported. If BreSeq is used, then a summary section of all major and low frequency minor variants will be included.

### SIGNAL ncov_tools

#### Additional in-depth quality control analysis

A software package called *ncov-tools* (https://github.com/jts/ncov-tools) was developed by Dr Jared Simpson of the Ontario Institute for Cancer Research (OICR, Toronto, Ontario, Canada) for further assessment of sequencing results through standardized quality control assessment. Its inclusion within SIGNAL allows assessment of sequencing run-level or sample-level contamination and quality issues, allowing application of bioinformatic mitigation strategies or laboratory re-sequencing. Within SIGNAL, *ncov-tools* is a submodule that can be run after the main functions. Like SIGNAL, *ncov-tools* is a snakemake workflow that requires its own configuration file; however, rather than having the user configure an existing template or run the package separately, SIGNAL will prepare the necessary results files as symbolic links and create a configuration file that is compatible with *ncov-tools* for seamless operation. If generated, FreeBayes results will be prioritized over iVar results. The extensive results and reports generated by *ncov-tools* are placed within their own directory within the overall SIGNAL results.

### Demonstration data

In addition to national contributions with PHAC through the Canadian COVID-19 Genomics Network (CanCOGeN) throughout the COVID-19 Pandemic, the SIGNAL team has contributed to routine surveillance within Ontario, Canada in collaboration with Public Health Ontario, Sunnybrook Health Sciences Centre, Shared Hospital Laboratory, Mount Sinai Hospital, Hamilton Health Sciences, and others as a member of the ONCoV Genomics Coalition. To demonstrate SIGNAL output and applicability, we re-examined a subset of a provincial surveillance dataset of 55 samples collected from April 30 to June 11, 2020 that underwent ARTIC3 PCR (https://github.com/artic-network/primer-schemes **)** and Illumina paired-end 250 bp sequencing using a HiSeq instrument. At the time of sequencing, all sequences passed quality control metrics for the submission of consensus genome sequences to the GISAID database. For the reassessment, SIGNAL v1.6.6 was used along with the following software versions: pangolin v4.3.1, pangolin-data v1.28, constellations v0.1.12, scorpio v0.3.19, and nextclade v3.7.4 with dataset tag 2024-06-13-23-42-47Z.

### Benchmarking data

We have also tested SIGNAL using a series of six benchmarking datasets that cover a variety of test cases including rapid and routine surveillance as well as the ability to identify both VOC and non-VOC lineages (20). Full results of the benchmarking can be found in Software and Data Availability. We will strive to update the benchmarks periodically as new features and updates to SIGNAL are released. For all cases, SIGNAL v1.6.6 was used, including the following lineage assignment software versions: pangolin v4.3.1, pangolin-data v1.28, constellations v0.1.12, scorpio v0.3.19, and nextclade v3.7.4 with dataset tag 2024-06-13-23-42-47Z.

## Results and discussion

### Major features of the workflow

High-throughput sequencing has become a critical tool for understanding the nuances of the evolving COVID-19 pandemic. However, handling increasingly large volumes of sequencing data is broadly challenging, whether due to bioinformatics inexperience or hardware capabilities and scale. SIGNAL provides a comprehensive workflow with detailed analysis and easy-to-access tools for the initial setup. Once SIGNAL is executed, the software manages all other dependencies, streamlining the experience for those with limited bioinformatics or computational skills. To address limitations in hardware accessibility, Snakemake also allows for optimized computer resource management and controlled software environments, which are employed to make efficient use of available hardware. During development, we provided optimized thread counts per Snakemake rule that provides optimal hardware conditions without diminishing returns or strain on the system while also lowering the minimum hardware requirements. While the use of Snakemake as an extension to Python makes SIGNAL accessible to a wide variety of systems, software compatibility issues within individual environments can result in mixed successes as updates for individual software tools can create conflicts. To minimize this, we standardized the software versions for the majority of individual environments. The notable exceptions are the lineage assignment tools PANGOLIN and NextClade, where the accelerated update schedules (often multiple times per week during the height of the Pandemic) of both required a step in the workflow to ensure that these lineage prediction tools, and their reference data were always up to date. Yet, in aid of reproducibility (and limited access to external internet resources), we added the option for the user to have full control of the lineage assessment software versions.

### Core output from SIGNAL

**Figure 1.**
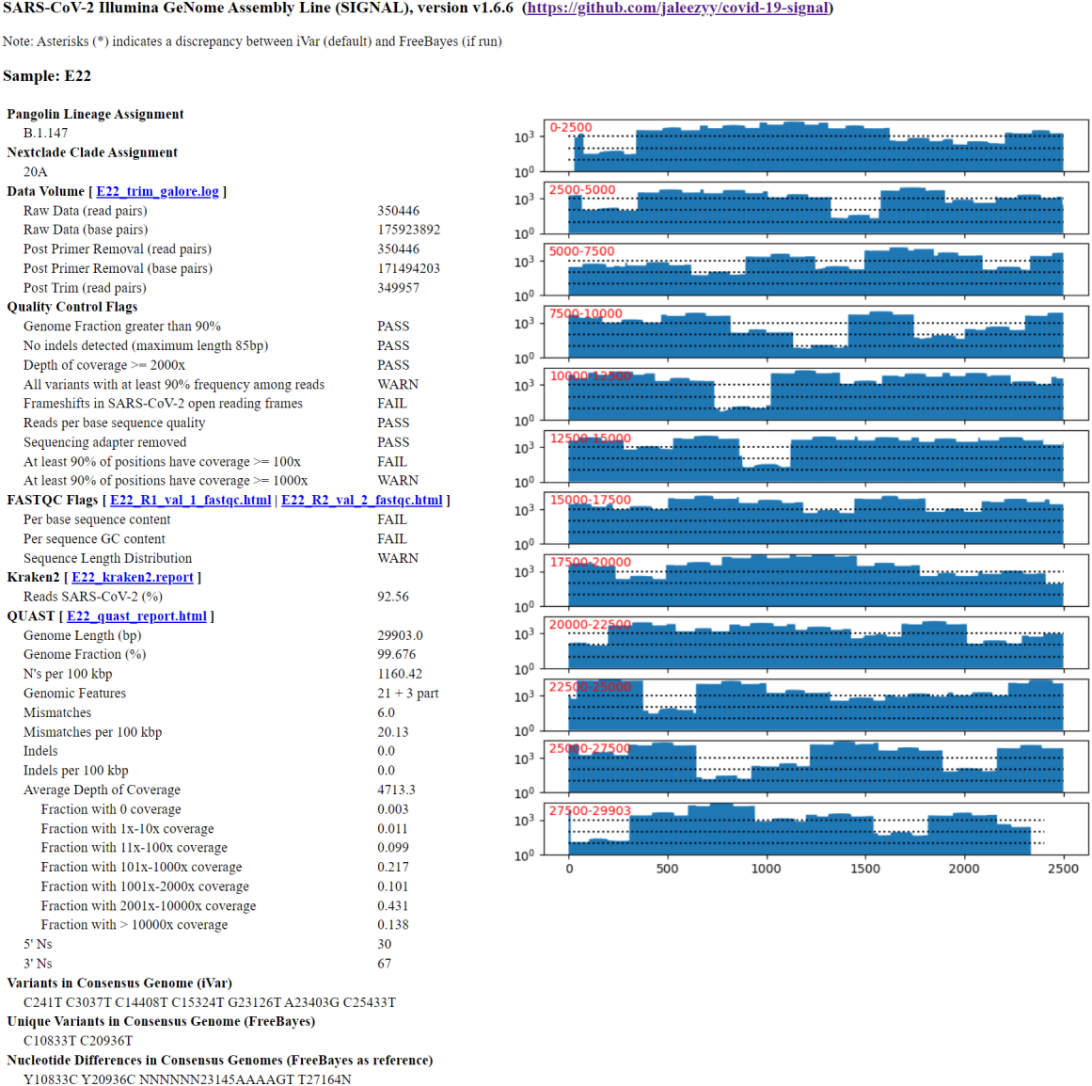
Example output of the individual sample report in HTML format. The lineage assignments are provided immediately to flag potential VOCs or interest. Quality control metrics are consolidated from several tools including FASTQC, Kraken2 and QUAST. Individual variants are highlighted from both iVar and FreeBayes (if the latter is used). Any differences in the assessment between iVar and FreeBayes consensus sequences will be indicated in the report. The text file version lacks the coverage plots, whereas the HTML format lacks a listing of BreSeq minor variants.

For each sample, a text and HTML file (Figure 1) is provided summarizing the key results. The assigned PANGO lineage and NextClade assignments are the first sections and quality control metrics follow, with a breakdown of raw data volume such as the number of reads pre- and post-trimming and filtering. Also, a verdict on the quality of the consensus genome is provided, including whether trimming was successful, whether the genome fraction is >90%, and whether the depth of coverage is >100×, 1000× or 2000×. A warning is provided if indels are detected as these genome sequences are often rejected by public databases. Select FASTQC parameters are also highlighted: ‘Per base sequence content,’ ‘Per sequence GC content’ and ‘Sequence length distribution.’ From Kraken2, the percentage of SARS-CoV-2-specific reads will be noted. Any additional viral reads not belonging to SARS-CoV-2 can be quickly identified as part of troubleshooting by their taxonomic origin, indicating potential contamination. Quality control of the consensus sequences from both iVar and FreeBayes (if present) are assessed using QUAST. Genome length and fraction values are reported along with terminal ‘N’ character counts and N’s per 100 kilobase pairs (kbps) overall. Mismatches and indels are counted and a breakdown of depth of coverage is provided. A summary of variants depicted as individual majority SNPs is provided for the iVar consensus genome relative to the specified reference accession (i.e. MN908947.3). The FreeBayes consensus sequence is also processed, and unique variants found in FreeBayes that are not found in the iVar consensus sequence are highlighted in a separate section. Furthermore, an alignment is performed between the iVar and FreeBayes consensus genome sequences using Parasail (21) and direct comparisons are reported using FreeBayes as the reference to report potentially spurious variants adjacent to indels when indel-realignment is not performed, as outlined above. Lastly, a set of coverage plots depicting read coverage across the SARS-CoV-2 genome are included in the HTML output only, while BreSeq variant calls are listed only in the Text output (although a link is provided to the BreSeq individual sample report if BreSeq was executed during analysis).

**Figure 2.**
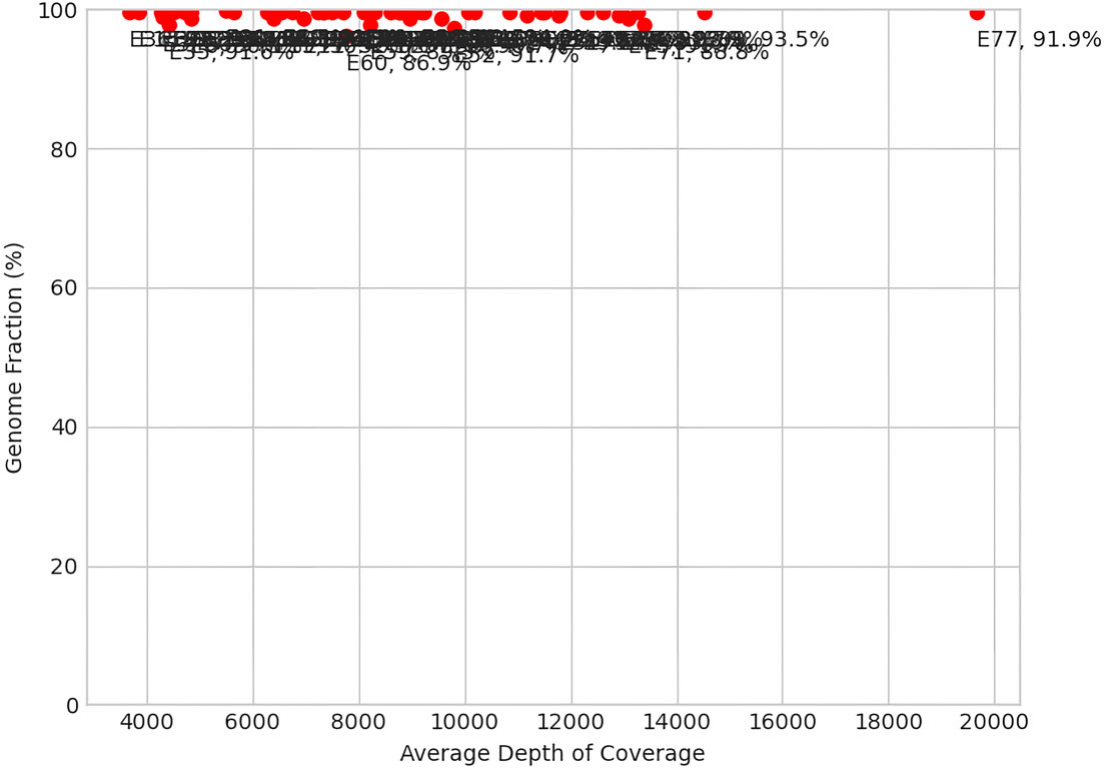
Example matplotlib output of percent genome fraction versus average depth of coverage across the sampled SARS-CoV-2 genomes.

**Figure 3.**
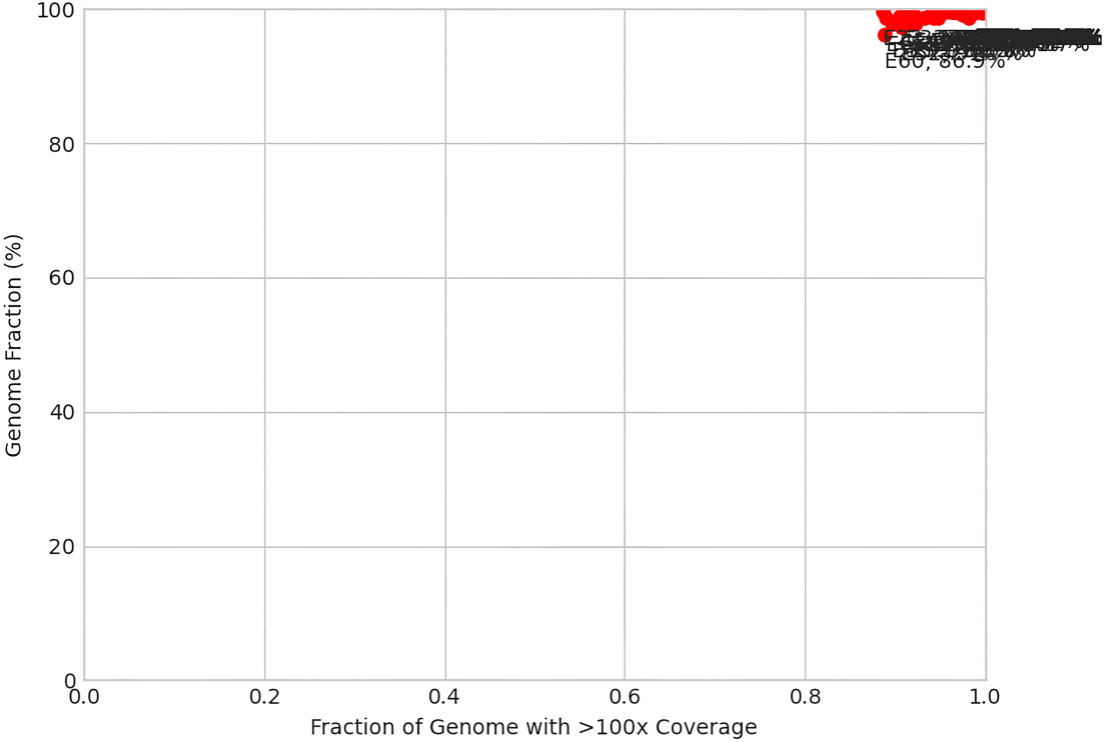
Example matplotlib output of percent genome fraction versus fraction of genome with >100× coverage across the sampled SARS-CoV-2 genomes.

**Figure 4.**
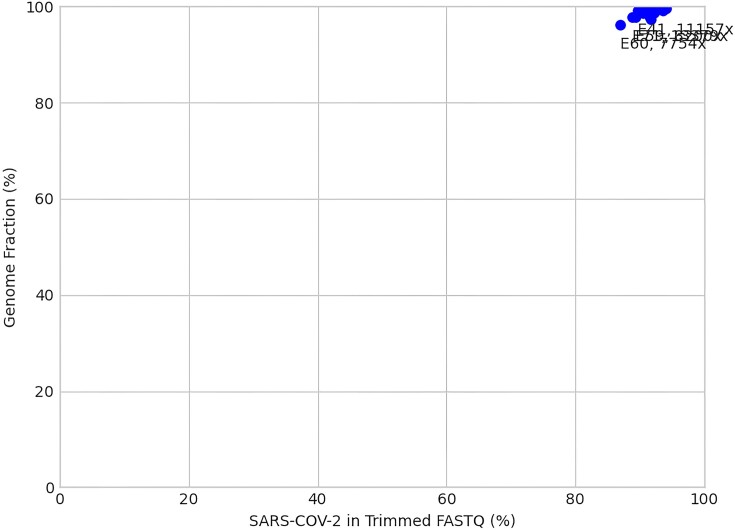
Example matplotlib output of percent genome fraction versus percent of trimmed reads from SARS-CoV-2 across the sampled SARS-CoV-2 genomes.

**Figure 5.**
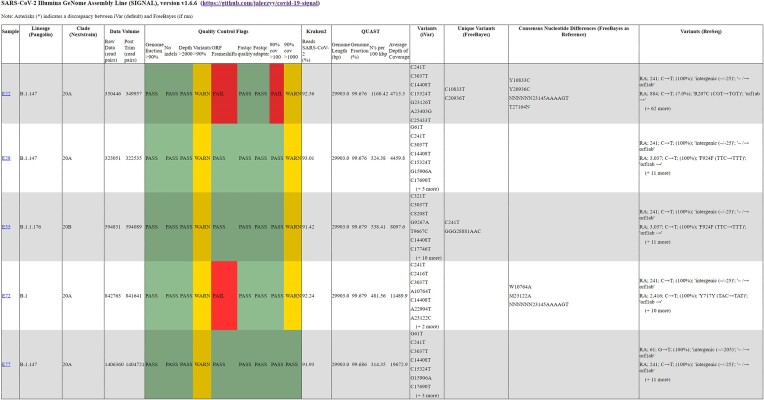
Example HTML format output consolidating the individual sample analyses (top 5 results plus negative control shown). A hyperlink is available within the ‘Sample’ column to access individual reports. The table produced may vary depending on the execution of optional tools such as BreSeq. Any discrepancies in lineage assignment (PANGOLIN and NextClade) found between iVar and FreeBayes consensus sequences are noted in their respective columns.

Beyond the reports for individual samples, three summary plots are produced based on coverage and genome fraction across all samples. The first plot depicts genome fraction against the average depth of coverage (Figure 2). Samples that do well will often demonstrate a higher genome fraction with a higher average depth of coverage across the SARS-CoV-2 genome. The second plot demonstrates similar trends with genome fraction against the fraction of the consensus genome with 100× or greater coverage (Figure 3), whereby samples that have read coverage >100× across the genome will produce a higher overall genome fraction. The third and final plot shows percent genome fraction against the percent of SARS-CoV-2 reads (Figure 4). Virus-rich samples (i.e. low *C*t values) with more reads attributed to SARS-CoV-2 often result in a higher genome fraction and cluster at the top-right of the plot, whereas negative controls and poorer quality samples (i.e. high *C*t values) will trend to the bottom-left of the plot. A complete summary HTML file is also generated which collates the individual sample statistics files into an easy to view format (Figure 5).

### Core output ncov-tools quality control

For a given run, a set of graphical plots and tables are produced summarizing the quality of the results in greater depth. Such output includes heatmaps depicting log10-scaled depths of coverage summarizing the abundance of sequencing data per-amplicon and identified SNPs, allowing for variant annotation (Supplementary Figures S2 and S3). SNP variation and PANGO lineage predictions are also summarized within a phylogenetic context, using the reference SARS-CoV-2 genome (i.e. MN908947.3) (Supplementary Figure S4). Text-based tabular output further reports ambiguously sequenced positions (Supplementary Table S1) and the presence of amplicons within negative controls (Supplementary Table S2).

### Benchmarking

A total of six benchmarking datasets covering a wide array of sequencing conditions were analyzed, including those generating failed metrics, lineage identification including VOIs and VOCs, outbreak analysis and routine surveillance. For benchmarking failing samples, SIGNAL will strive to attempt a lineage prediction but was unable to do so for 20 of 24 (83.3%), i.e. agreeing with the benchmark, yet was able to predict lineage for 4 ‘failed’ samples, in three cases due to iVar's better handling of frameshift mutations and in one case due to higher tolerance for long stretches of unresolved (N) nucleotides. For VOI and VOC identification, SIGNAL was able to match 100% of the predictions across the 16 representative samples in the benchmark dataset. Conversely for non-VOI and non-VOC identification, 5 out of 39 (12.8%) representative samples failed to produce a matching lineage prediction. An expected B.1.1.431 prediction was reported as B.1.1 and an expected B.1.1.391 prediction was reported as B.1.1.450. Three samples with expected lineages of B.1.2, B.1.450 and B.1.515 were all categorized as the B.1 lineage. However, the benchmarking datasets were based on samples and lineage predictions from 2021 and SIGNAL defaults to using the latest versions of both PANGOLIN and Nextclade lineage calling software. The discrepancies observed in the non-VOI and non-VOC datasets may be attributed to updated reference data used by SIGNAL. A unique feature of SIGNAL is the ability for users to define versions of software including PANGOLIN, Nextclade and their associated lineage data. The ability to alter versions allows for replication and for updates to past analyses using more current references.

### Operational performance and limitations

SIGNAL consolidates several established tools of the trade with regards to processing DNA sequencing data. All the individual tools are well-established in their performance and accuracy. No alterations are made to any of the individual tools and instead the significance of SIGNAL is its optimization of each tool’s implementation towards an overall objective analysis. Speed and performance of SIGNAL was one of our top priorities in its initial development at the start of Canada's first wave of cases. In practice, recurring waves would drive an increased number of samples for rapid and accurate assessment of lineages, novel mutations and VUIs/VOCs. SIGNAL’s performance can be gauged by comparison to other published SARS-CoV-2 sequencing workflows, including PipeCoV (5), which benchmarked across 120 samples for PipeCoV, QIAGEN CLC Genomics Workbench (CLC) (https://digitalinsights.qiagen.com/products-overview/discovery-insights-portfolio/analysis-and-visualization/qiagen-clc-genomics-workbench/), V-pipe (22), ViralRecon (23,24) and SIGNAL. All of the pipelines in this benchmark assessment, including SIGNAL, have been publicly available as early as June 2020 and all have played important roles in processing SARS-CoV-2 sequencing data. ViralRecon (23,24) is a Nextflow workflow part of the nf-core framework that supports both Illumina short-read and Oxford Nanopore long-read data. V-pipe (22) is another Snakemake workflow aimed at solely processing Illumina short-read data that use unique profile hidden Markov models (HMMs) that are tailored to small, diverse viral genomes. CLC (by QIAGEN) offers a graphical user interface for performing a wide variety of bioinformatics workflow analyses for SARS-CoV-2 short- and long-read sequencing data. PipeCoV (5), unlike SIGNAL and the other workflows mentioned, uses Docker containers combining of reference-based and *de novo* assembly methods to capture genomic variation while preserving the biological structure including single/multiple nucleotide variants and indels. Our benchmarking analysis was completed in 1 h and 53 min using SIGNAL compared to the second fastest ViralRecon at 19 h and 52 min, with minimal tradeoff in accuracy when using reference-based methods, which is significant for deployment of SARS-CoV-2 surveillance efforts. Notably, the incorporation of BreSeq into SIGNAL increases execution time. While low frequency minor variant data may not be needed for population surveillance, where tracking the prevalence of consensus lineages is the focus and thus BreSeq is excluded in SIGNAL’s default settings, they can be used as a powerful tool to track co-infection and evolution of the virus within individual patients (25,26).

### Limitations and conclusions

One of the main limitations of SIGNAL is its exclusivity to Illumina paired short-read sequencing data. Long-read technologies such as PacBio and Nanopore continue to improve with further advancements, and we can see examples of application towards assembling coronavirus genomes (5,27,28). Similarly, SIGNAL is highly specialized in its requirement of Illumina short-reads such that alternate short-read forms such as IonTorrent (29) would require modifications specifically for preprocessing of the sequencing reads. The second main limitation is SIGNAL’s optimized focus on SARS-CoV-2 assembly only. Given the initial emergence of SARS-CoV-2, a novel coronavirus, genomic initiatives were focused solely on its detection and subsequent alterations; however, these initiatives have since expanded to include other viral pathogens. To address this limitation, users are given the ability to manipulate parameters put forth for analysis, including the reference genome and primer schemes, which allows for reference-based assembly and general analysis of viruses other than SARS-CoV-2. However, due to the pipeline being optimized for SARS-CoV-2, lineage assignment functionality will be limited as both PANGOLIN and Nextclade exclusively assign lineages to SARS-CoV-2 within SIGNAL. Nextclade has since released several additional datasets allowing for assignment of pathogens other than SARS-CoV-2, including several lineages of influenza virus, respiratory syncytial virus (RSV) and measles, among a growing collection. Future versions of SIGNAL will expand the available Nextclade datasets available to the pipeline to address this functionality. Overall, SIGNAL is an easy-to-deploy, rapid and accurate SARS-CoV-2 analytical pipeline for one of the most prevalent sequencing technologies currently available. Its ease-of-use closes gaps for application of SARS-CoV-2 genomic surveillance in a diversity of settings.

## Supplementary Material

lqae176_Supplemental_File

## Data Availability

SIGNAL v1.6.7 source code is available at Zenodo (DOI:10.5281/zenodo.14194675). In addition, version controlled updates are available at GitHub (github.com/jaleezyy/covid-19-signal). All SIGNAL output files for the re-analyzed and benchmarking samples are also available on Zenodo (DOI:10.5281/zenodo.14194677) and GitHub (github.com/jaleezyy/signal-example-data). All corresponding sequencing reads for reanalysis have been deposited in BioProject PRJNA689621. All consensus genome sequences and associated metadata in this dataset are published in GISAID’s EpiCoV database: to view the contributors of each individual sequence with details such as accession number, Virus name, Collection date, Originating Lab and Submitting Lab and the list of Authors, visit DOI:10.55876/gis8.240628bf.
