# Supplementary material for: SARS-CoV-2 Illumina GeNome Assembly Line (SIGNAL), a Snakemate workflow for rapid and bulk analysis of Illumina sequencing of SARS-CoV-2 genomes
Source: NAR Genom Bioinform. 2024 Dec 18;6(4):lqae176. doi: 10.1093/nargab/lqae176 (PMC11655287; doi:10.1093/nargab/lqae176)
Supplement: lqae176_Supplemental_File [file lqae176_supplemental_file.pdf]

Supplementary Table 1. Example output of ambiguous positions from a single sample, generated by ncov-tools. High counts of alternate alleles suggest an inconsistency across samples that may be due to contamination.

Position	Count	Alleles
21	3	Y
61	7	K
529	3	K
586	4	Y
643	5	Y
913	11	Y
955	3	Y
1059	10	Y
1154	6	R
1347	10	Y
1541	10	Y
1554	4	R
1900	3	M
1943	5	Y
1947	20	Y
2659	4	K
2836	3	Y
2781	3	R

Supplementary Table 2. Example output of the negative control report, generated by ncov-tools for a single sample. High prevalence of amplicon sequences (ARTIC3) in negative controls could suggest cross-contamination between positive and negative control samples. Note, the tab-delimited text report gives the full name and path for the underlying BED files but is simplified in this table.

File	QC	Genome Covered Bases	Genome Total Bases	Genome Covered Fraction	Amplicons Detected
Plate3BLANK1	WARN	588	21090	0.028	
Plate3BLANK2	WARN	1388	21090	0.066	6,50
Plate3BLANK3	WARN	5160	21090	0.245	
Plate4BLANK1	WARN	7267	21090	0.345	44,62,75,87,93
Plate4BLANK2	WARN	13100	21090	0.621	3,42,44,46,47,53,58,62,69,70,72,79,95
Plate4BLANK3	WARN	14148	21090	0.671	33,44,46,47,53,58,75,79
Plate4BLANK4	WARN	11056	21090	0.524	14,22,25,30,34,46,47,53,62,75,82,87,93

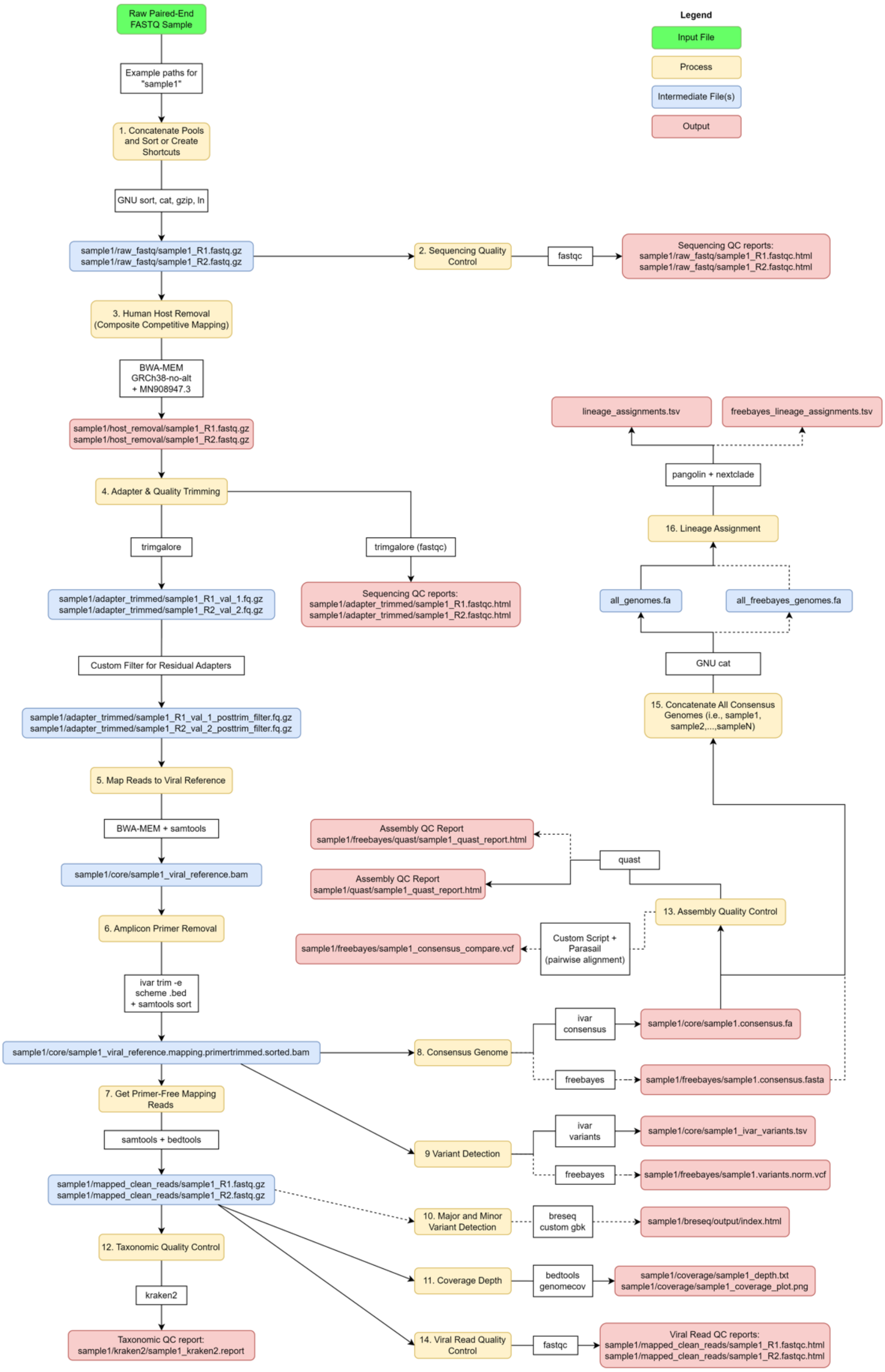

Supplementary Figure 1. Schematic of the SIGNAL workflow (*signalexe.py all*). From paired short-read input, the workflow is executed per-sample in parallel. Trimming steps are applied to remove human-host and non-viral reads to ensure only reads that align to the SARS-CoV-2 genome (i.e., MN908947.3) are used for reference-based consensus generation, variant calling, lineage assignment, and quality control steps.

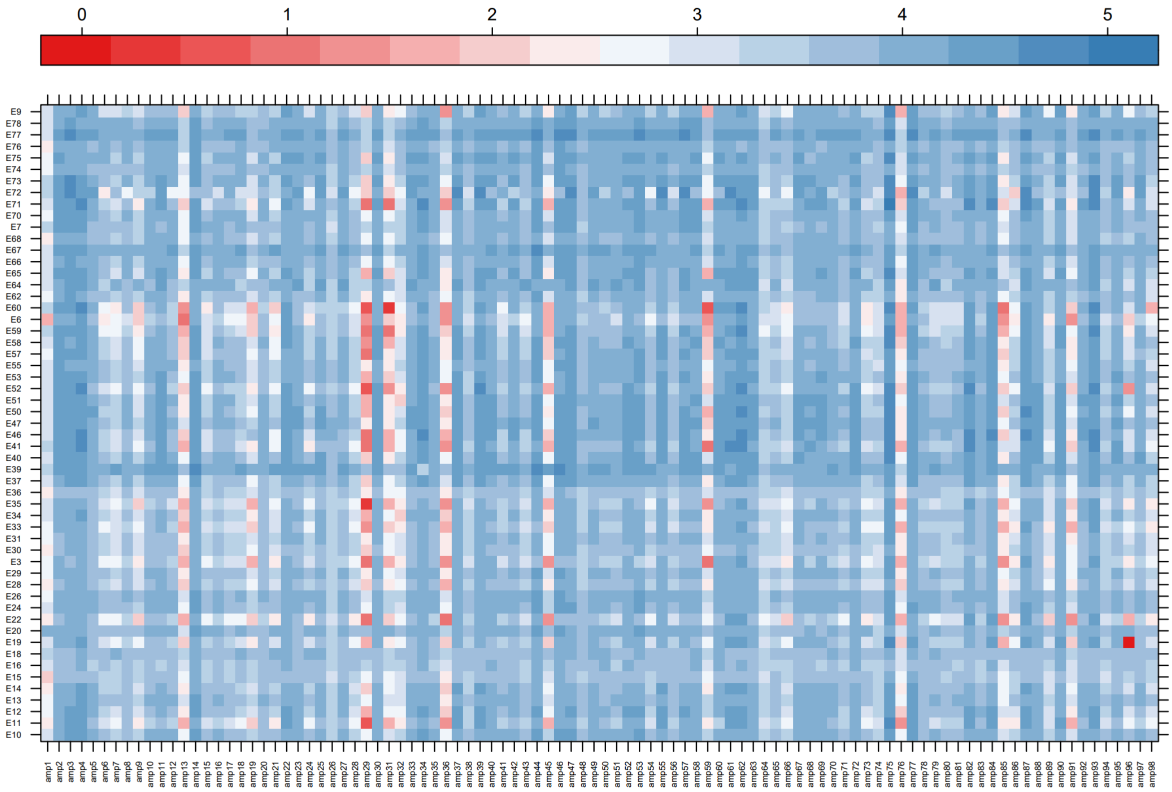

Supplementary Figure 2. Example heatmap output showing log10-scaled amplicon coverage, generated by ncov-tools. The horizontal axis lists all the amplicon identifiers and the vertical axis lists all samples.

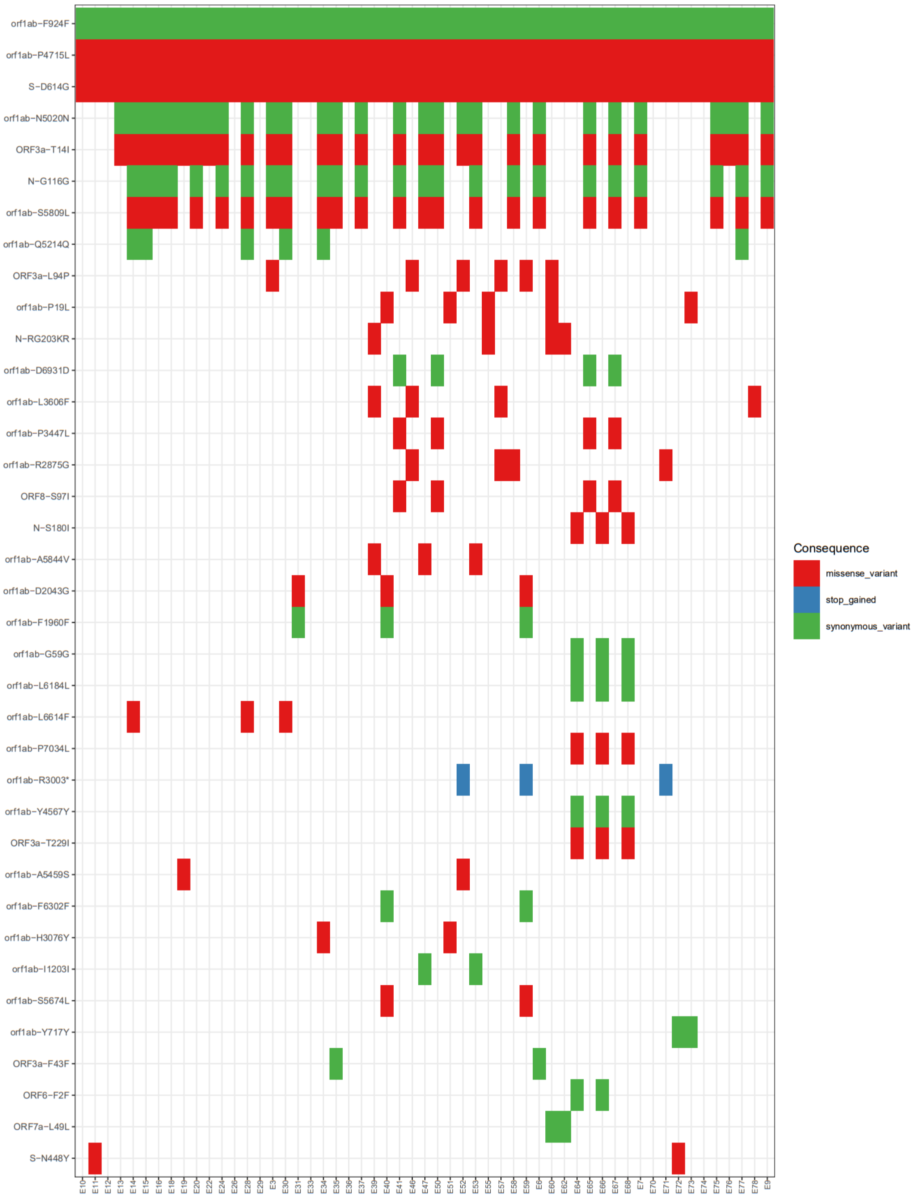

Supplementary Figure 3. Example ncov-tools output showing annotated variants. The horizontal axis shows all samples and the vertical axis lists all mutation-gene combinations.

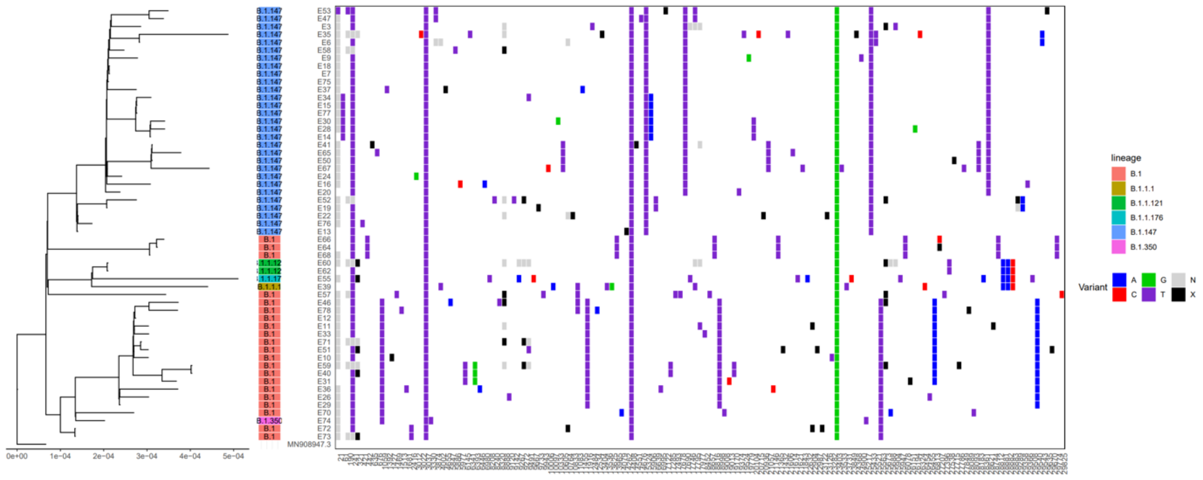

Supplementary Figure 4. Example ncov-tools output showing variation across consensus sequences using a phylogenetic tree with associated single nucleotide polymorphism (SNP) matrix. The SARS-CoV-2 genome reference (i.e., MN908947.3) is represented at the bottom with the horizontal axis depicting genome position. Coloured tiles in the SNP matrix depict the variant found at a given position.
